# Developing a gist-extraction typology based on journalistic lead writing: A case of food risk news

**DOI:** 10.1016/j.heliyon.2018.e00738

**Published:** 2018-08-20

**Authors:** Youngkee Ju, Myoungsoon You

**Affiliations:** a School of Communication, Hallym University, 1 Hallym Daehak-Gil, Chuncheon, 24252, Republic of Korea; b Department of Health Science in the Graduate School of Public Health, Seoul National University, Seoul, 08826, Republic of Korea

**Keywords:** Education, Information science, Public health

## Abstract

Risk communication is challenging since scientific knowledge is likely to be targeted to the public, which may have inadequate knowledge to understand jargons and expertise in risk messages. This study aims to construct a journalistic gist extraction typology, which can be useful for developing risk messages. Journalists' lead writing was conducted for 164 governmental press releases regarding food risks, and they were compared to factual information in original press releases. seven types of gist extraction were identified: ‘exemplifying,’ ‘contextualizing,’ ‘grouping,’ ‘identifying likely victims,’ ‘emotional appeal,’ ‘separating verbatim,’ and ‘sense-making numbers.’ The typology was valid with 92% of the total leads made by nine reporters being applicable to it. The content analysis revealed that ‘exemplifying’ was the most frequent gist extraction type, followed by ‘contextualizing’ and ‘separating verbatim.’

## 1 Introduction

It is well known that a news lead should tell the reader “the gist of the event” and report a story's essential facts in “a brief, sharp statement” (Charnley, 1966; Mencher, 2010). Journalism has established its own guidelines and ideal patterns for covering news, and lead writing is also likely to be a subject of the discipline. Even though this journalistic discipline is supposed to be a basis for quality journalism that represents reality in an accurate, simple, and clear way, this journalistic pattern of lead writing has rarely been applied for developing messages other than news stories.

It is also recognized that we live in a “risk society”, wherein hazards and insecurities induced by modernization are dealt with systemically (Beck, 1992; Lupton, 1999). Thus, efficient risk communication methods that connect expert knowledge to the coping behaviors of the general public has taken on increased importance. A challenge when developing risk-communication messages is ensuring that jargon and expert knowledge are phrased clearly and simply so that the general public can understand the message. Therefore, risk communicators can be considered as “the interpreters, clarifiers, and simplifiers of technical jargon” (Ng and Hamby, 1997, p. 474). Taking this challenge into account, government institutions addressing safety issues (e.g. the Food and Drug Administration, Center for Disease Control and Prevention, Nuclear Regulatory Commission, Canada Public Health Association) as well as international organizations (e.g. the World Health Organization, European Food Safety Authority) consistently emphasize the need to craft “simple” messages that are easy to understand. The Nuclear Regulatory Commission, for example, encourages risk managers to “tailor messages to an easy level of comprehension,” use “simple short sentences,” and make “comparisons to put risks in perspective” (Persensky et al., 2004).

This study examined how news leads capture the gist of events and attempts to construct a typology in journalistic lead writing to make non-journalistic messages easier to comprehend by eliminating unfamiliar jargon, concepts, and complex science while retaining the essential message. We first examined the psychological perspective of how memory develops and is used in information processing, known as fuzzy-trace theory (FTT), which suggests that people prefer retrieving gist memories to retrieving verbatim memories, implying that gist-centered messages are more effective for processing risk information. We then constructed a gist extraction typology for which journalistic lead writing was used as a basic source, since this type of writing is generally based on gist extraction.

This study consisted of three stages: development, validation, and application for gist-extraction typology. In the first stage of typology development, we asked newspaper, TV, and internet news channel reporters to write leads for press releases concerning food risks. A former journalist with a 10-year reporting career also wrote leads. Reporters commented on why they constructed the leads as they did. After the lead data were collected, the content of lead writings and the comments were closely reviewed to identify journalistic decision-making patterns, which led to the compilation of a typology. In the validation stage, we tested the validity of this typology by asking the reporters to determine if each of the leads that other reporters made on the food risks belonged to one of the gist extraction patterns in the typology provided to them. Finally, in the application stage, we analyzed actual news coverage to evaluate how frequently each type of gist extraction was used in news coverage conveying risk information.

Although gist itself is considered as a type of memory that is studied in psychology, how to extract gist has rarely been examined. Thus, this study is unique in that it developed a gist extraction typology using journalistic lead writing, and applied it to actual news coverage. Developing a typology of news leads in terms of gist extraction can also enrich the educational content of journalism in addition to being useful for developing efficient risk communication strategies, especially for those that involve crafting effective educational messages.

### 1.1 Gist and verbatim in information processing

Understanding how to communicate complicated messages involves an understanding of how people process information, which includes understanding how memories develop. According to FTT (Reyna and Kiernan, 1994), there are two types of memory that trace experiences: gist trace and verbatim trace. Gist trace refers to “an abstract representation of semantic content that does not incorporate details of surface form” (Reyna and Kiernan, 1994, p. 180). A more concrete representation of information that incorporates the details of the surface form is called a verbatim trace. The surface form here indicates that verbatim information is located in a memory target that individuals experience. For example, if three rectangles, A, B, and C, of sizes 30 cm, 20 cm, and 10 cm, respectively, are placed on a table from left to right, the verbatim memory for this case would dictate that the sizes of A, B, and C are 30 cm, 20 cm, and 10 cm, respectively.

On the other hand, memory would also recognize that A is larger than B, and B is larger than C. In a more abstract way, this situation could be remembered as objects becoming smaller from left to right. Compared to the memory that directly articulates the specific size of the three objects, the latter two examples are more abstract based on an individual's interpretation, or gist extraction, and therefore represent gist-based information. Thus, gist is an abstract representation of semantic, relational, and other elaborative information and is *interpreted* and *added* to a memory target by individual interpreters.

As a theory of memory development, FTT addresses critical issues concerning how gist and verbatim information are processed, stored, and retrieved. First, psychologists suggest the “parallel storage of gist and verbatim trace”, which implies that both types of information are encoded and stored simultaneously during information processing (Brainerd and Reyna, 2004; Broniatowski and Reyna, 2017). People are able to remember that A is 30 cm and C is 10 cm. At the same time, they remember that the objects become smaller from left to right.

FTT also suggests that there could be multiple kinds of gist-based memory that depend on interpretation or gist extraction. The final example, which involved remembering that the rectangles were smaller “from left to right,” reflects a greater degree of gist extraction compared to the second example that compared each pair of rectangles. We can therefore say that there are different levels of gist extraction.

Last, and more importantly, FTT suggests the preference of gist over verbatim in decision-making. When individuals perceive a target and store it in their memory, they develop verbatim memory, interpret the verbatim information, which we call gist extraction, and thereby also develop gist memory (Brainerd and Reyna, 2004). However, when individuals make decisions, they are more likely to activate gist than verbatim, i.e., the fact that the three objects become smaller from left to right would be preferred over recalling that the length of A was 30 cm and the length of C was 10 cm (Reyna and Kiernan, 1994). Risk communication usually seeks to change attitudes or behaviors, so developing gist-based messages would be expected to be important for successful risk communication. In other words, appropriate gist extraction is critical for efficient risk communication.

### 1.2 Gist extraction and journalistic lead writing

Gist extraction is a typical method of information processing, and, for the general public, multiple types of gist can be developed during information processing (Reyna and Brainerd, 1995). However, the extraction of the gist from stimuli is an area that has received little empirical investigation, particularly since gist has tended to be studied as a type of *memory* rather than a factor to be considered in message development (Brainerd and Reyna, 1990, 2004; Reyna, 2008). Here, we posited that certain gist-extraction patterns may be more appropriate for communicating complicated information. The examples of effective gist extraction strategies can be observed from an examination of lead stories written by reporters who have been trained to capture and convey the gist in a simple and clear message. Journalism has established its own guidelines, principles, and/or ideal patterns for covering news. Given these patterns or standardized forms for reporting and news writing, we anticipate that extracting gist from complicate information at the level of risk-message developers, for example, can also benefit from the development and use of a gist extraction typology.

To substantiate this perspective on the use of a gist extraction typology, a closer examination of the conceptual aspects of both gist extraction and journalistic lead writing is needed. A lead should tell the reader “the gist of the event” or “the gist of a completed action” (Warren, 1951; Bush, 1954). In this respect, lead writing requires gist extraction, as ordinary people do when they are exposed to risk information. However, gist extraction through lead writing and by audience reading have slightly different foci. The psychological notion of gist is defined as an abstract representation of an object that does not incorporate detail (Reyna and Kiernan, 1994). Here the focus is an *abstractness* based on the extraction. The paper by Reyna and Kiernan was not concerned with whether gist represented an essential part of a target object but instead focused on how multiple types of gist can be extracted from observing an event. However, for lead writing an essential part of the story is emphasized, and many introductory books on news writing explain that an effective lead “captures the essence of the event” (Mencher, 2010, p. 111) or is “a brief, sharp statement of the story's essential facts” (Charnley, 1966). Therefore, the “gist of the event” in lead writing focuses on the essential part of the event, unlike ordinary people's gist extraction, characterized by the abstractness of an interpretation.

This raises the question: does journalistic lead writing actually represent the psychological notion of gist as an abstract representation of an object? We posit that the lead itself is likely to be abstract in one way or the other when its conceptual aspect in journalism is considered. The lead as a representation of an essential part of the main text should be written as a general expression that represents a set of news information covered in detail in a later part of the main text. Therefore, it is reasonable to expect the lead to be abstract when it generally represents in one sentence multiple pieces of relevant information that is later explained using multiple sentences. Consequently, we can say that the lead is likely to be as abstract as gist extraction, but is more likely to represent the gist of a story than does ordinary gist extraction. A reporter's gist, in other words, is likely to be closer to the content essence than gist extracted by ordinary individuals.

Bearing these similarities and differences in mind, we recognized that journalistic lead writing could be a reference point for gist extraction in communication. In journalism education, many lead typologies are presented, and they explain the type of lead that works best for a given story and audience. For example, summary leads, anecdotal leads, descriptive leads, question leads, quote leads, and direct-address leads are suggested as the paradigm lead formats (Yopp et al., 2010). Many other categorization types are also provided, and explaining all of them in detail is beyond the scope of this study. The point here is that most lead type categories are not determined based on types of gist extraction. Quote leads, for example, indicate the lead form, regardless of whether the content represents the gist. Although summary leads are only concerned with capturing the most important point, they do not originate from the results of an empirical study of gist extraction, either.

Taking the above information into account, we attempted to directly explore and identify the types of gist extraction in lead writing. This empirical investigation of journalistic lead writing can specify how the essential meaning of a news story is extracted, and thereby assist in designing effective communication that has specialized jargon and expert knowledge. We addressed our first question:

**RQ1:** What kinds of gist extraction techniques are applied to journalistic lead writing to report risk information?

The first research question was examined by identifying the repeated patterns of gist extraction. Based on the results from the first question, we then asked a second question regarding how often each type of gist extraction was used in actual news coverage:

**RQ2:** Which types of gist extraction are frequent in actual news story leads that cover risk information?

## 2 Methodology

The data for this study are unique as well as complicated in that incumbent reporters were asked to write the leads for food risks after reading actual press releases published by a governmental public health organization. They were also required to evaluate the gist-extraction typology developed in terms of its applicability to each lead made by another reporter. IRB approval for having the reporters write leads and evaluate the gist-extraction typology was obtained from Seoul National University Institutional Review Board (49-2013-07-25). We also collected the informed consents from the participating reporters. The content of actual news coverage that was previously published was analyzed as well. Therefore, this complicated methodology may be better summarized in terms of the three stages: typology development, validation, and application.

### 2.1 The typology development stage

News releases by a Korean governmental public health office and news leads about the news releases written by Korean reporters were collected. Regarding the type of message to investigate, we selected food risk as a topic because it is considered to be an issue “with universal interest” (Anderson, 2000, p. 255). Developing gist extraction typology from lead writing was first attempted in this study and we were not sure at that point whether gist extraction regarding a specific issue can represent gist extractions for all issues universally. In this circumstance, we choose an ordinary health risk, food risk, as an initiating study of gist extraction.

Government press releases by the Ministry of Food and Drug Safety (MFDS) in South Korea were utilized as a major source of information about food-related health hazards. In total, 212 news releases were posted online from July 5, 2013 to February 5, 2014. Of these, we excluded 48 news releases that were not related to food or drug hazards, or those that announced administrative policies and activities with no specific link to a particular hazard. A total of 164 news releases were selected as sources for the reporters' lead writing.

To identify a journalistic gist extraction typology, nine reporters who covered the MFDS participated in writing leads for 27 or 28 press releases concerning food risks. They also provided several sentences explaining why they wrote the lead as they did. The reporters' ages, journalism career length (yr), and types of news media are presented in Table 1.

**Table 1:** Career information for reporters who participated in lead writing and validity check.

	News Media	Age (yr)	Reporting experience (yr)
Reporter1	National Daily	42.0	15.0
Reporter2	National Daily	47.0	20.0
Reporter3	National Daily	28.0	1.7
Reporter4	TV	46.0	20.0
Reporter5	TV	39.0	15.0
Reporter6	TV	48.0	20.0
Reporter7	News Agency	35.0	8.0
Reporter8	News Agency	46.0	20.0
Reporter9	Internet News Media	34.0	8.0

Together, the leads and the journalists' explanations for them served as resources to construct a typology of gist extraction. To develop the typology, a former reporter with a 10-year reporting career closely reviewed the reporters' leads, comments, and relevant news releases by MFDS to compare and find identifiable patterns that were repeated in the leads. In particular, this reporter scrutinized the relationship between the factual information and expressions in the lead sentences with those in the entire text of each press release. We predicted that the comparison of the news releases as news source and the leads as supposedly the essential gist of the news releases could be used to identify observable and repeated patterns and these repeated patterns could become a type of gist extraction.

### 2.2 The typology validation stage

Since the emerging typology would be the first attempt to represent journalistic gist extraction, other data from the reporters were collected to validate the typology. A few weeks after the original lead writing, the nine reporters who had written the leads were asked to check the validity of the typology by determining whether each of the leads could belong to one of the gist-extraction types developed and, if so, choosing the type that best fits the case of the lead under investigation. To help the reporters determine the applicability of the gist-extraction types to actual news stories, operationalization and examples of each type were provided to the reporters, and detailed information of each gist extraction type is explained in the Results section. Each reporter examined 27 or 28 leads made by another reporter who participated in the lead writing.

The abovementioned process for validation by the reporters was to determine how much the typology could or could not be applied to actual leads made by incumbent reporters. We posit that the ecological validity can be strengthened by the current study design in which incumbent reporters who cover food risk actually write the leads for certain food risks and evaluate another reporter's leads in terms of the gist-extraction typology developed from their own lead writing. The reporters also rated each gist extraction type in terms of perceived usefulness and frequency of actual use in their news writing. A 7-point Likert scale was used for the survey (1 = “never,” 7 = “very useful”/“very frequently”).

### 2.3 The typology application stage

In addition to validation, we also attempted to apply the typology to actual news coverage of food risk. For this application, news media stories relevant to each of the 164 news releases were collected through Korea Integrated News Database System (KINDS), a Korean news website that represents 65 news media outlets, including 10 national daily newspapers and 3 nationally broadcast TV stations. This collection is to check how frequently the gist extraction typology developed is applied to actual news coverage. News stories distributed nationwide were investigated by limiting the content analysis to 10 national daily newspapers and 3 TV stations. News stories were identified as relevant to a given news release if they mentioned the name of the MFDS and the hazard covered in each news release.

Stories published over a seven-day period- from three days before to three days after the date of the original news release- were examined. News stories released before the online appearance of the press release were included because the MFDS may have distributed press releases to journalists before they were posted on the MFDS website. For news stories released before the original press release, the content of the news coverage was checked to see if the stories indeed concerned the press releases under investigation. News stories that did not address a given issue in the MFDS press release, even though it mentioned the same hazard, were excluded. In total, 321 news stories disseminated by the 10 national daily newspapers and 3 TV stations that were related to the 164 press releases were collected.

Two coders read the collected news stories, with one reading all articles and the other reading 50 randomly selected articles (13.5%). The same operationalization used for the incumbent reporters was used. The inter-coder reliability was .72 (Scott's pi). Chi-Square tests were conducted to determine whether each gist-extraction type had significantly different frequencies.

## 3 Results

### 3.1 Journalistic gist-extraction typology

The first and main question of this study focused on the types of gist extraction that are applied to journalistic lead writing of risk information. A scrutiny of reporters' explanations on why they constructed the leads in the ways they did, and a comparison of press releases and news leads identified seven gist extraction types: ‘exemplifying,’ ‘contextualizing,’ ‘grouping,’ ‘identifying likely victims,’ ‘emotional appeal,’ ‘separating verbatim,’ and ‘sense-making numbers.’

The exemplification was the type of gist extraction first identified. This type of extraction occurred in a lead where the most salient, familiar, or noticeable example was presented in order to extract the gist from the conveyed news information (Table 2). When a risk message regarding fine dust particles gave various safety tips, for example, recommendations that masks be used or contact lenses be avoided appeared in the lead. Thus, a mask or contact lens was the *familiar* item that was used to guide readers/listeners to the essential message that the fine dust particles were harmful and individuals can and should do something to prevent the hazard from affecting them. This perceived pattern was also supported by the reporters' comments including: “Kimbab (a traditional Korean food), cold noodles, and salad were included in the lead because they are major foods subject to *Escherichia coli* contamination,” “I selected the most noticeable one among the multiple tips for preventing food poisoning,” “the most significant part was exemplified to present the items for pre-announcement of legislation,” and “I selected aspermia since it seemed the most significant side effect.”

**Table 2:** A typology of journalistic gist extraction and its operationalization.^1^

Type of Gist Extraction	Operationalization	Examples and Specification
Exemplifying	When a risk message involves multiple pieces of information that concern a similar object, an item of the greatest intensity, familiarity, or attainability is put into a lead	• “Glasses are preferred to contact lens when fine dust particles are prevalent.” (When various safety tips concerning fine dust particles were suggested)
Contextualizing	When a risk message involves jargon and expertise or other unfamiliar risk factors, relevancy-intensifying information such as use, purpose, or reason is added to a lead.	• “Four materials that can be misused as drugs are newly regulated by the anti-drug act.” (When the press release reported that “Fluoroamphetamine” and three other materials were newly subject to governmental regulation by the anti-drug act.)
Grouping	When a risk message involves multiple pieces of information represented by jargon, they are integrated and represented by a common characteristic or category explained by familiar words or those coined by reporters.	• Five products represented by brand names including “KCF Baking Chicken Powder ” were categorized as “baking powder” • When a single jargon is additionally explained by a familiar category pertaining to the jargon, it is considered as contextualizing rather than categorizing • “With the fall season beginning, which is characterized by a large daily temperature range, the public health institution has issued food poisoning watch.” (When the press release delivers varieties of information for preventing food poisoning)
Identifying likely victims	The identity of a group exposed to a risk or a target group for a health policy is placed in a lead.	• “Sales and advertising of high-caffeine drinks to children is regulated.” (When the law was revised to prohibit sales of high-caffeine drinks at schools)
Emotional appeal	The likelihood of death, or the name of serious/infectious disease is referenced. Safety-related phrases such as “dangerous,” “serious side effect,” or “being safe” is directly addressed in a lead.	• “The food that contains ephedrine, which causes insomnia, or even hypertension was recalled.” (The press release just mentioned that inedible food was recalled, with its side effect being explained later.)
Separating verbatim	When verbatim of jargon, expertise and proper nouns are mixed with gist in the first paragraph of a press release, gist is separated and placed in the lead, while the remaining verbatim appears later in the article.	• “The minced garlic made at an unregistered place was recalled.” (The information on name of producer, product, and inspector was omitted.)
Sense-making numbers	Making sense of quantitative information is conducted by rounding, summarizing, visualizing, providing maximum/minimum, or comparing to a sum, average or earlier amount in a lead.	• “The number of patients from Norovirus that constitutes more than half of food poisoning in winter is increasing.” (When the number of 504 <53%> was provided) • “Honeyed Red Ginseng Tea that had passed the expiration date by more than three years was recalled.” (The information on the hazardous products that had passed their expiration date by a few months to a few years was provided.) • “The government crackdown on adulterated food during Thanksgiving holidays revealed that one out of ten makers violated the Food Sanitation Act”. (The press release reported that 2,127 businesses were inspected and 197 were found to violate the law.)

1 We consider demonstrating the process of the various gist extraction types to be the most valuable property of the study. Therefore, a richer set of examples is provided in the Appendix.

Providing context for less-comprehensible jargon was identified as another gist extraction method. For example, the additional explanation about the purpose or use of an unfamiliar product relevant to a certain hazard represented by the jargon was a typical case of contextualization. In this case, both the product and its negative effect are unfamiliar, so people may have difficulty processing the message. Providing simple contextual information can therefore guide the gist of a message about a particular hazard. Some exemplary comments by the reporters supporting this type of gist extraction were “urushiol was hard to understand, so I added that it is dangerous because it is likely to cause dermatitis,” “the reason for recalling Chinese Kimchi was added,” “I additionally explained the place where the turmeric powder containing excess lead was sold,” “a reason was added after the fact to help understand the information at a glance,” and others.

For grouping, a few reporters said that “the name of the material was so unfamiliar that I did not mention it even as an example, and it was just referred to as a *kind of drug*,” or “could not list the specific names of all the components, and simply referred to them as ‘particular material’”. Exemplifying and contextualizing can be comparatively less-complicated gist-extraction operations in that an example is *selected* to represent multiple items, or a familiar explanation regarding the purpose, reason, use or other contextual information relevant to the risk is added. Grouping, however, can be regarded as higher-level gist extraction as it requires the integration of multiple objects by similarity or under a particular criterion to create a new category or a higher level of abstraction. This type was not frequently observed in either reporters' lead writing or news coverage.

A more appropriate example of grouping can be found in the lead saying “MFDS released tips for discerning toadstools and emergency measures in order to reduce accidental consumption of poisonous mushrooms.” The relevant press release addressed various aspects of toadstools such as their characteristics, how other mushrooms can be difficult to distinguish from toadstools, whether poison in the mushroom can be destroyed by heat, post-consumption symptoms, recommended measures after the symptoms appear, and general guidelines for Fall mushroom hunting. One can see that these various types of information were integrated into “tips for discerning toadstools” and “emergency measures” in the lead. The specific operationalization for this gist extraction is presented in Table 2.

Identifying likely victims was also a gist-extraction type in the leads. When a risk was likely to affect particular groups, the gist information regarding this risk involved information about who or what groups might be affected. For example, when a spice powder imported from Bangladesh was found to contain lead in excess of allowed quantities, the lead sentence was: “A brand of spice powder imported from Southeast Asia and distributed to foreign workers was banned for sale as it exceeded the standard allowed lead quantity.” Reporters themselves mentioned that “I considered that the main buyers of the spice powder were East Asians,” “emphasized the small restaurant owners as they were major recipients of the benefit,” and so on. Although small restaurant owners who receive particular benefits reported in the press release are not direct victims, we considered this situation as an example of victim identification, given that restaurant owners could also be affected.

In the case of emotional appeal, fatal aftermaths or serious risk implications were placed in the lead sentence, thereby likely eliciting fear or other negative emotions by addressing the worst-case scenario. For example, the MFDS investigated 22 diet food products sold online, and found that three used toxic substances. A lead highlighting the worst possibility was written as follows: “Some diet foods on an online market were banned as they contained toxic substances that could cause cancer, myocardial infarction, or depression.” Zajonc (1984) suggested “the primacy of affect” in which the affect comes to mind faster than cognitive association. This is similar to the ideas of “hot cognition” (Abelson, 1963), “affective memory” (Arnold, 1970), and “affective tag” (Fiske and Pavelchak, 1986), all of which support the earlier activation of affect. Even though a risk message involves specialized jargon and expert opinion, which could complicate comprehension and recollection, the emotional appeal can help the reader/listener understand the gist of the warning.

In addition, there were the situations in which the gist of a risk message was successfully extracted from the first paragraph of the press release. Reporters can use gist by just omitting the additional verbatim text. This extraction type, therefore, would be the most convenient type for reporters, as would be expected considering that ideal press releases are inherently customized for media.

In general, there were three levels of ease in the aforementioned gist extraction: the most convenient method was separating the gist from the verbatim text, while the least convenient were grouping, which requires an integration of the text. The others were less complicated, as instead of needing to integrate multiple kinds of information, only specific information, such as an example, contextual information, likely victims, or emotion-arousing aftermath, were selectively addressed.

Risk message gist extraction can also involve quantitative information. For example, when food poisoning by the norovirus caused 53% of all total food poisoning cases, a numeric simplification such as “more than half of food poisonings” was used in a lead. This extraction is referred to as “sense-making numbers” (Table 2). In essence, risk information is categorical whether it is dangerous or not, and most technical information regarding a risk involves quantitative information, so readers may have difficulty determining the degree of danger. Sense-making a number in this respect can assist the audience in grasping the categorical implications of the risk at hand.

When multiple numbers were involved in a risk message, a salient characteristic of the risk was shown by exemplifying the utmost point of the numeric information or comparing the quantitative text to a reference number. In the first case, numeric risk information was represented by the largest, smallest, or the total number, while in the second case, the numeric text became more meaningful when compared to a reference. For example, a standard point for a food product suggested by the government can be a typical reference. Both methods were also gist extraction based on sense-making the numeric information.

### 3.2 The validity of the typology

Each reporter wrote 27 or 28 press releases and the nine reporters wrote 246 leads in total. Thus, even though the total number of press release was 164, we had each reporter write leads for more than 1/7 of all press releases in order to increase the sample size as much as possible based on the consideration of the ordinary workload of journalists. Out of the total 246 leads, only 19 cases (7.72%) could not be assigned to the typology, while the remaining 92.28% fell into one of the defined categories.

Furthermore, when the reporters were asked if each gist extraction type was useful for ordinary news writing, the average response was a 5.56 (*SD* = 0.79) on a 7-point Likert scale, with 7 being “very much so” and 5 being “tend to be so.” When it comes to “frequent use in ordinary news writing,” the average response was 5.33 (*SD* = 0.89) on the same scale. The data and evaluation indicated that the typology had more than 92% applicability to the reporters' actual lead writing and was evaluated to be useful for working journalists to write leads.

In addition, it is also worth noting that young reporters with less career experience reported less frequent use and considered the typology less useful, indicating ‘4’ or ‘5’. A reporter from an internet news channel, characterized by less rigorous journalism standards, also showed a similar pattern. This implies that journalists with more career experience and those with more journalistic training are more likely to feel the typology useful and report that they use it frequently. This may be another validation of the typology.

### 3.3 Salient types of the journalistic gist extraction

**Fig. 1:**
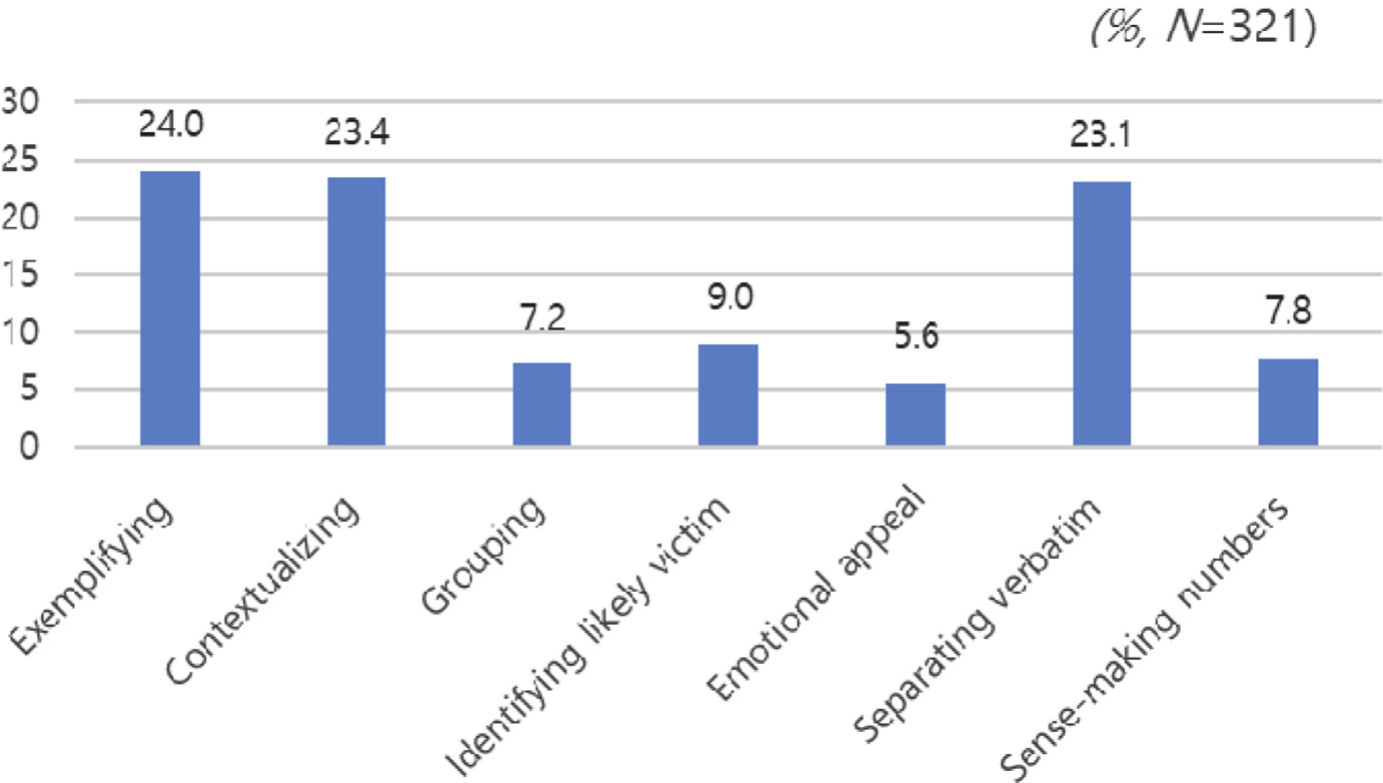
Frequency of each gist extraction type found in South Korean news coverage of food risk (July 5, 2013–February 5, 2014).

The second research question sought to determine the gist extraction method most favored in actual news coverage, which was examined by quantifying previously published news stories. Of the total 321 news stories disseminated by 10 national daily newspapers and three TV stations, the exemplifying type (77) appeared the most frequently, followed by contextualizing (75), separating verbatim (74), identifying likely victims (29), sense-making numbers (25), grouping (23), and emotional appeal (18) (Fig. 1).

## 4 Discussion

In the present study, nine incumbent reporters participated in lead writing and their leads were used to identify the journalists' gist extraction patterns. As a result, seven types of gist extraction were identified: exemplifying, contextualizing, grouping, identifying likely victims, emotional appeal, separating verbatim, and sense-making numbers. The seven types of gist extraction were evaluated by the reporters as being applicable to 92.28% of the 246 leads they had made. When the frequency of each extraction type in actual news coverage was quantified, exemplifying was the most frequent, followed by contextualizing and separating verbatim.

We developed a gist-extraction typology based on journalistic lead writing and this outcome belonged to neither content analysis study nor message effect research. In this respect, this study is unique in terms of its practical nature. This uniqueness, however, does not imply a lack of theoretical connection between this study and early communication studies. A few types of gist extraction appeared to be consistent with findings of previous studies. For example, exemplified risk information was effective (So et al., 2017), and Zillmann (2006) summarized research reports on the effects of exemplifying presentations on safety and health beliefs. For gist extraction by contextualizing, which refers to providing additional, and relevancy-strengthening information for an unfamiliar risk message, Petty and Cacioppo (1979) found that people tend to take a closer look at evidence if the message has a greater degree of personal relevance. These examples signify that the two types of gist extraction used by the journalists would be effective in communicating messages. A more comprehensive examination of previous studies that are relevant to each type of gist extraction may provide further insight.

In addition, it should be noted that gist extraction does not always happen in the lead, although the present study assumes that gist extraction in lead writing should be informative for general message construction. In some stories that are not about breaking news, the lead serves more to draw audience attention than to convey the gist of a story. Therefore, gist extraction and lead writing should not be equated in the real world of journalism. This recognition opens a new area of study: an analysis assessing how much certain leads conduct gist extraction. In other words, leads may extract gist in different degrees and communication studies can delve into investigating the differences in lead writing per se and the effect of the differences of gist extraction.

This line of research can lead to theorizing about journalistic lead writing by combining the psychological notion of *gist extraction* and empirical data from journalism. Whereas ideal types of news leads are presented in a normative sense by many journalism textbooks, to our knowledge there has been no empirical examination of lead writing in terms of how gist is extracted from all available information for a news event. We believe that this study can be an initial point from which further studies can be developed to determine efficient, theoretically-supported gist extraction methods and their effects. In this study, exemplifying, for example, was much more prevalent than grouping or sense-making numbers. Although we assume that journalistic gist extraction is more likely to be efficient than that of ordinary individuals, we do not know whether these favored types are more effective in communication than the less-favored types. Additional work on this issue will deepen the understanding of gist extraction and its role in news consumption.

This study also has significance in terms of psychological gist research. Gist and verbatim have previously been studied only as a type of memory in psychology rather than as a type of message. Whereas memory belongs to a later part of information processing, we focused on the earlier part, gist extraction, which has rarely been studied.

This study does have some limitations. Although it suggests a typology of journalistic gist extraction, the study results are based on risk news coverage focused on a particular type of risk (food risk) in a particular country (South Korea). Investigating journalistic gist extraction methods in other regions with different types of news stories would thus be valuable to assess the reliability of the developed gist extraction typology.

## Declarations

### Author contribution statement

Youngkee Ju: Analyzed and interpreted the data; Wrote the paper.

Myoungsoon You: Conceived and designed the experiments; Performed the experiments; Contributed reagents, materials, analysis tools or data.

### Funding statement

This work was supported by the Ministry of Education of the Republic of Korea and the National Research Foundation of Korea (NRF-2015S1A3A2046760), and Hallym University [HRF-201801004].

### Competing interest statement

The authors declare no conflict of interest.

### Additional information

No additional information is available for this paper.

## Appendix A Supplementary data

mmc1 mmc1
