# Supplementary material for: Developing a gist-extraction typology based on journalistic lead writing: A case of food risk news
Source: Heliyon. 2018 Aug 20;4(8):e00738. doi: 10.1016/j.heliyon.2018.e00738 (PMC6104525; doi:10.1016/j.heliyon.2018.e00738)
Supplement: mmc1 [file mmc1.docx]

**Appendix.** Examples of the seven types of journalistic gist extraction.

| Type of Gist Extraction | The 1^st^ paragraph of the press release | The leads | Note |
| --- | --- | --- | --- |
| Exemplifying | - The Ministry of Food and Drug Safety announced that it had inspected 20 kid cafés in order to prevent food poisoning. As a result of inspecting the various locations in Busan City, Ulsan City, and South Gyeongsang Province from November 4 to 6, 13 out of 20 cafés were found to have violated the food safety act and the MFDS asked the local governments to issue fines/penalties accordingly. | - 590 day-old salt and pepper were found in the kitchen of "A" Kid Cafe in Dongrae-gu, Busan, bought when the cafe first opened. Such condiments and spices were used on pork cutlets and pizza dishes that children subsequently consumed. Cleaning of the kitchen was so rarely done that grease spots on the ceiling were spotted. | - Sanitary inspections of over 20 kid cafés were conducted and the press release reported the results. The newspaper selected one extreme case and put it on the lead. This case accurately depicts the unsanitary surroundings of the 13 establishments caught during the inspections. |
| Contextualizing | - The Ministry of Food and Drug Safety announced that US beef processed at SWIFT BEEF CO. was twice found to contain Zilpaterol (0.35ppb and 0.64 ppb out of 22 tons of beef, respectively), and thereby banned from entering the domestic market, stopping further imports to Korea. | - Beef imported from the US was found to contain substances prohibited from use. Zilpaterol is a hormone injected into cows for faster growth. | - Zilpaterol was the matter of concern but it was not familiar to the public. Contextual information on its use was provided along with its description as a prohibited substance. |
| Grouping | - MFDS announced that it will provide safety tips that help people buy gifts (health foods, medical appliances, etc) for *Chuseok^[[1]](#footnote-1)^* in a safe way. * This press release contains safety information on various activities that take place during the holidays (cooking, gift shopping, frequent use of nausea medication for excessive driving to hometown, etc.). | - As *Korean Thanksgiving* *(Chuseok)* drew nearer, an "assorted safety information set" covering gift shopping, cooking, taking nausea medication, etc. was released. | - Various tips on multiple safety issues during the *Chuseok* holiday was grouped into an 'assorted safety information set'. This grouping requires an abstraction over various factual information |
| Identifying likely  victims | - The MFDS announced that since November, it started to provide risk information regarding hazard substances in foreign food and drugs, including cosmetics and medical devices via mobile texts specifically geared towards smartphone users. This press release later explains the background for this new service, being related to the increase of international travel and online shopping, through which foreign products are directly delivered to users. | - Information on foreign hazardous food products is provided via smartphone for international travelers and online shoppers. | - This information is useful especially for international travelers and online shoppers, and these target groups are included as gist information. |
| Emotional appeal | - MFDS announced that the health food Mi Sam Jung (containing ginger products), made by Myunsung-sa in Changwon city were found to contain aconitine, an ingredient of a plant that is prohibited from being used in health food items. The products were recalled and additional sales were prohibited. *The effects of aconitine were explained during the press release. | - Health foods containing a substance that incites respiratory failure and/or paralysis were recalled. | - The worst case side effect was added to extract gist from the press release, which conveys details including the product name, manufacturing company, company location etc. |
| Separating verbatim | - MFDS announced that mozzarella cheese produced by Fonterra Co. in New Zealand was found to contain *Staphylococcus aureusthe,*bacteria that elicit food poisoning. The bacteria were discovered in 2 cases out of 232 tons imported, one made in January 18th, 2013 and the other on January 29th, 2013. All products were subsequently recalled. | - Pizza cheese from New Zealand was found to contain food poisoning bacteria and was recalled by the company | - The gist of the message in the press release was all conveyed in the first paragraph. Therefore, non-essential information (e.g, amount, time, name etc.) was excluded. |
| Sense-making  numbers | - MFDS announced that it recalled *daegu* *jeon^[[2]](#footnote-2)^* products distributed by Kumho Trade Co. (located in Gimpo city) as the company arbitrarily extended the expiration date of the products. The recalled fillets originally expired in August 31st, 2015. The company extended the date up to 307 additional days. | - Cod fillets with expiration dates arbitrarily extended up to more than 10 months were recalled and prohibited from further sales. | - "307 days" were simplified into "more than 10 months" to help the readers recognized the seriousness of forging the expiration dates of food products.    |

1. Korean Thanksgiving [↑](#footnote-ref-1)
2. a Korean cod fillet dish [↑](#footnote-ref-2)
